# pH-Responsive hyperbranched polypeptides based on Schiff bases as drug carriers for reducing toxicity of chemotherapy†

**DOI:** 10.1039/d0ra01241f

**Published:** 2020-04-06

**Authors:** Rui Yan, Xinyi Liu, Junjie Xiong, Qiyi Feng, Junhuai Xu, Haibo Wang, Kai Xiao

**Affiliations:** College of Biomass Science and Engineering, Sichuan University Chengdu 610065 China whb6985@scu.edu.cn; National Chengdu Center for Safety Evaluation of Drugs and National Clinical Research Center for Geriatrics, West China Hospital, Sichuan University Chengdu 610041 China xiaokaikaixiao@scu.edu.cn

## Abstract

Polymeric micelles have great potential in drug delivery systems because of their multifunctional adjustability, excellent stability, and biocompatibility. To further increase the drug loading efficiency and controlled release ability, a pH-responsive hyperbranched copolymer methoxy poly(ethylene glycol)-*b*-polyethyleneimine-poly(*N*ε-Cbz-l-lysine) (MPEG-PEI-PBLL) was synthesized successfully. MPEG-PEI-NH_2_ was synthesized to initiate the ring-opening polymerization of benzyloxycarbonyl substituted lysine *N*-carboxyanhydride (*Z*-lys NCA). The introduction of Schiff bases in the polymer make it possible to respond to the variation of pH values, which cleaved at pH 5.0 while stable at pH 7.4. As the polymer was amphiphilic, MPEG-PEI-PBLL could self-assemble into micelles. Owing to the introduction of PEI, which make the copolymer hyperbranched, the pH-responsive micelles could efficiently encapsulate theranostic agents, such as doxorubicin (DOX) for chemotherapy and NIRF dye DiD for *in vivo* near-infrared (NIR) imaging. The drug delivery system prolonged the drug circulation time in blood and allowed the drug accumulate effectively at the tumor site. Following the guidance, the DOX was applied in chemotherapy to achieve cancer therapeutic efficiency. All the results demonstrate that the polymer micelles have great potential for cancer theranostics.

## Abstract 2

Polymeric micelles have great potential in drug delivery systems because of their multifunctional adjustability, excellent stability, and biocompatibility.

## Graphical abstract

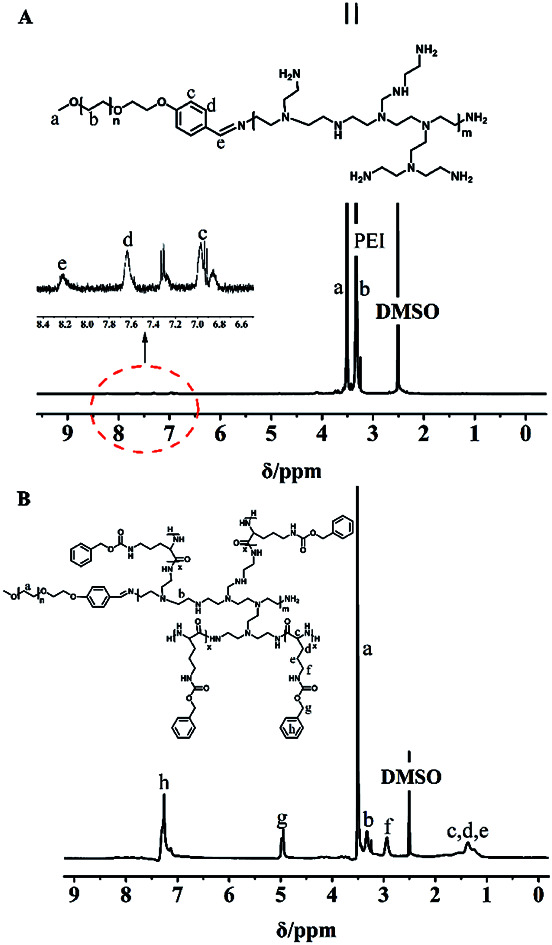

## 1. Introduction

Significant advancements have been made in cancer therapy. Polymeric micelles are considered as one of the most effective means with a promising prospect to effectively improve the pesticide effect and reduce the side effect to the normal cells.^1–7^ Despite the bright prospects they have, there are still some challenges need to be overcome, such as controllable drug release behavior, degradable in the body and good biocompatibility, and high drug loading capacity.^8–12^ Without these characteristics, it is difficult to achieve the ideal therapeutic effect and even possible to cause some side effects in cancer therapy.^13–15^

It is known that tumor cells have many specific features different from normal cells, such as high glutathione concentration, glycolysis with lactate secretion, low pH level, and so force, which enable researchers to treat cancer with controllable and efficient approaches.^16–22^ Among them, the pH-oriented studies have attracted many attentions, as tumor cells provide energy mainly through anaerobic respiration, while the acid–base environment in normal tissues and bloodstream is neutral, the pH value in tumor tissues is ranging from 4.5 to 6.5. The pH gradient makes it feasible to design pH-responsive polymeric vehicles that can release drug at tumor site in control. Researchers endow the polymeric micelles pH responsiveness *via* introducing acid-sensitive covalent bonds.^23–29^ Among them, Schiff base is often used as pH-responsive linker in polymer chains. Jin *et al.*^30^ constructed a triblock copolymer *via* the reaction between aminooxy terminals of oxime-tethered polycaprolactone (OPCL) and aldehyde-terminated PEG (PEG-CHO), which can be used as drug carrier responsive to the acidic microenvironment. Wang *et al.*^31^ selected the polymer PEG-*b*-PLKC and 4-(decyloxy) benzaldehyde (DBA) as the reactants to prepare the Schiff base, the benzoic imine bond is broken when the pH is reduced to 6.5 but retrieved as the acidic environment became neutral.

As reported in previous study, undegradable polymers used for disease treatment *in vivo* will result in a series of syndrome like malignancy inducted by frustrated phagocytosis and prolonged inflammation.^32,33^ To address this problem, biodegradable materials have attracted a growing attention to construct drug delivery system (DDS). Polypeptides, possessing the similar structure to proteins, are unique biodegradable and biocompatible synthetic polymers, meanwhile, they are stable against hydrolysis while rapidly biodegrade into α-amino acids *in vivo* in the presence of specific enzymes. Moreover, with the ring opening polymerization (ROP) of virous *N*-carboxyanhydride,^34–37^ it is facile to tune the ratio of hydrophilicity and hydrophobicity segment and the property of the polymer. The use of polypeptide-based polymer in DDS has been widely reported. Liu^38^*et al.* designed a cholesterol-decorated poly(l-cysteine) copolymer whose assemblies could transform from micelle to vesicle in response to reactive oxygen species (ROS). Ji^39^*et al.* developed several photoresponsive biodegradable PEG-*b*-PNBG diblock copolymers, which have many potential applications in cancer therapy. Although nano polymeric micelles based on biodegradable materials have shown many inspiring advantages, in practice poor drug loading capacity usually limits the actual application of them, which make the treatment useless or cannot meet the desired effect.^40–42^

Notably, hyperbranched polymer, the structure of which could provide stable cavity improving the drug loading capacity,^43,44^ has been developed to enhance the drug loading capacity. In this work, a pH-responsive copolymer based on Schiff base consisting of PEG, polyethyleneimine (PEI) and polypeptide chains was synthesized and studied as polymeric micelles. PEG was widely used as a gold standard in drug delivery system although recent research revealed that it may lead to immunoreaction.^45^ On one hand, hyperbranched polyethyleneimine (PEI) was introduced in the polymer to connect with PEG through Schiff base linkages to initiate ring opening reaction of lysine NCA. Due to the hyperbranched structure of the polymer,^46^ the drug loading content of the polymer micelles was up to 25.8%. On the other hand, the benzyloxycarbonyl substituted polylysine^47–49^ was chosen as the hydrophobic block, which was facile to obtain the prospective degree of polymerization and was naturally biocompatible with the structure close to protein. Moreover, the theranostic agents like DOX and near-infrared dye DiD were encapsulated into the pH-responsive micelles effectively. The morphology, drug loading and release profiles of the micelles *in vitro* were investigated. Furtherly, *in vivo* cellular uptake, biocompatibility, and pharmacokinetics were investigated to evaluate the biology performance of the MPEG-PEI-PBLL micelles. It is anticipated that this pH-responsive drug delivery system can greatly enhance the drug delivery efficiency and therapeutic outcome.

## 2. Experiment section

### 2.1. Materials and characterization

*N*ε-Carbobenzyloxy-l-lysine (*Z*-Lys, Macklin, 98%), *p*-toluenesulfonyl chloride (TsCl, Macklin, 99%), 4-hydroxy benzaldehyde (Macklin, 98%), triphosgene (Adamas, 99%), methoxypolyethylene glycols (MPEG, *M*_n_ = 2000 g mol^−1^, adamas), hyperbranched polyethyleneimine (PEI, *M*_n_ = 1800 g mol^−1^, adamas), doxorubicin hydrochloride (DOX·HCl, Adamas, 98%), the solvents THF, CH_2_Cl_2_ and DMF were used after expulsion of water. MPEG_2000_-C_6_H_4_CHO, and *Z*-Lys NCA were synthesized in advance referring to the previous articles.^50,51^

**Scheme 1:**
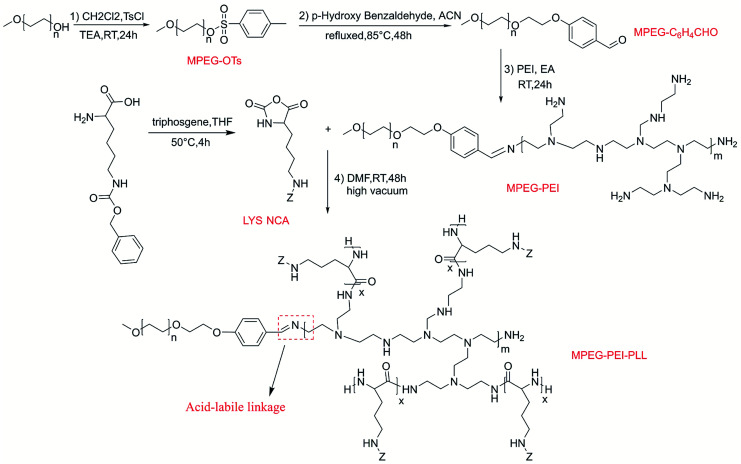
Synthetic route of MPEG-PEI-PBLL.

^1^H NMR spectra were recorded on a Bruker AV 400 spectrometers with DMSO-*d*_6_ as solvent. Fourier transform-infrared (FTIR) spectrometer (Nicolet 560 infrared spectrometer) was used to record FTIR spectra at 25 °C, and the transmittance mode was used. Transmission electron microscopy (TEM) images were acquired from a JEM-1200EX microscope. Dynamic light scattering (DLS) was measured on Malvern Nanozetasizer to analyze the diameters and distribution of the micelles. The concentration of DOX was measured by UV-visible absorption spectra in absorbance mode against a standard curve and PerkinElmer LS-55 spectrofluorometer was employed to measure the fluorescence spectra (Scheme 1).

#### Cell culture

Two non-small cell lung cancer cell lines (95D, A549) and one normal cell lines of rat cardiac cells (H9C2) were used in this study. 95D and A549 cells were cultured in RPMI 1640 (Biological Industries, Israel) supplemented with 10% FBS (Biological Industries, Israel) and 1% penicillin/streptomycin (Biological Industries, Israel). While H9C2 cells were cultured in Dulbecco's Modified Eagle's Medium (DMEM, Biological Industries, Israel) containing 10% FBS and 1% penicillin/streptomycin. Cells were incubated at 37 °C with 5% CO_2_ and 100% humidity. All cells were purchased from the Chinese Academy of Science Cell Bank for Type Culture Collection (Shanghai, China).

#### Animal and tumor xenograft model

All animals (female BALB/c nude mice, 6–8 weeks age) were purchased from Beijing Vital River Laboratory Animal Technology (Beijing, China). These animals were kept under pathogen-free conditions according to AAALAC guidelines. All animal experiments were approved by the animal experiments ethical committee of the Sichuan University ethics committee. To establish a subcutaneous xenograft model of lung cancer, 7 × 10^6^ 95D cells in 100 μL were injected into the right flank of the mice.

### 2.2. Synthesis of MPEG-PEI

MPEG_2000_-C_6_H_4_CHO (0.4 g), polyethyleneimine (PEI, 0.36 g) were dissolved in ethyl alcohol (50 mL) followed by 24 hours stirring at 25 °C. Then the solution was recrystallized at −20 °C overnight and filtered to get the product. The recrystallization process was repeated twice to get the final product followed by vacuum drying at 30 °C one day, white product (MPEG-PEI) was obtained (yield 0.56 g, 73%).

### 2.3. Synthesis of MPEG-PEI-PBLL

The copolymer was synthesized inside a round-bottom flask under high vacuum by ROP reaction. Typically, MPEG-PEI (0.3 g), *Z*-Lys NCA (0.3 g) were dissolved in DMF (8 mL), followed by 48 hours stirring at 25 °C under high vacuum and precipitating in diethyl ether. White solid was obtained after vacuum drying at 30 °C for 1 day (yield 0.45 g, 75%).

### 2.4. Preparation of blank micelles

Typically, MPEG-PEI-PBLL (10 mg) was dissolved in DMSO (2 mL), deionized water (10 mL) was added dropwise under vigorous stirring at room temperature. Then the aqueous solution was dialyzed against deionized water for 72 h in a dialysis bag (MWCO 3500 Da). The blank micelles were stored at −20 °C after lyophilization.

Diameter of the micelles were measured by Nano-ZS at 25 °C, TEM was introduced to characterize the morphologies of blank micelles. And critical micelle concentration (CMC) was measured using pyrene fluorescence probe method referring to the previous article.^52^

### 2.5. Loading of theranostic agents

DOX and DiD loaded micelles were prepared the same way. Typically, DOX·HCl (8 mg), MPEG-PEI-PBLL (20 mg) and a drop of TEA were dissolved in DMSO (5 mL), followed by stirring at 25 °C overnight. Afterwards, under vigorously stirring, deionized water (50 mL) was added slowly dropwise, dialysis and lyophilization were performed in turn as the same as blank micelles.

Drug loading content (DLC, wt%) and drug loading efficiency (DLE, wt%) were determined by UV absorption at 485 nm. The results were calculated as follows.
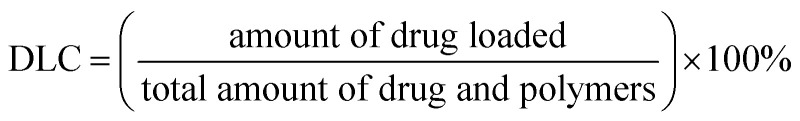

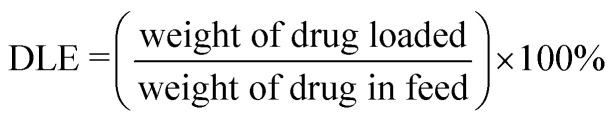


### 2.6. *In vitro* drug release

The drug loading micelles were incubated at different acidic environment in PBS using dialysis method to investigate release behaviors *in vitro*. The micellar solution (5 mL) of the polymer (1 mg mL^−1^) was dialyzed against 200 mL PBS (pH 7.4, 6.0, 5.0) in a rotary shaker at 37 °C. At set intervals, micellar solution (0.2 mL) was withdrawn and the amount of DOX remaining was calculated according to the result of UV absorption. Accumulative release of DOX from the micelles was calculated as follows.
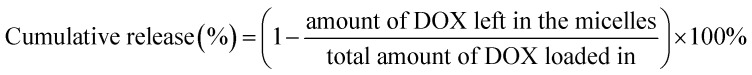


### 2.7. Cell viability assay

Cell viability was evaluated using the Cell Counting Kit-8 kit (Dojindo, Japan). Cells were plated in 96-well culture plates for 24 h and incubated with a series of concentrations of DOX (0–0.5 μM), MPEG-PEI-PBLL and DOX-loaded micelles (at an equivalent of 0–0.5 μM DOX) for 72 h. The cells were further treated with Cell Counting Kit-8 solution diluted to 10% with pure culture medium. After incubation for 2 h, the absorbance values were assessed at 450 nm with a reference at 650 nm using a microplate reader (Bio-Tek, USA). Every well was measured three times.

### 2.8. *In vivo* cell accumulation

After digestion and counting, 95D cells were seeded at a density of 3.0 × 10^5^ cells per well in six-well cell culture plates. After 24 h, DOX, or DOX-loaded micelles at 5 μM DOX per ml were added into the wells and the cells were incubated for 0.5, 3, 6, or 9 h, respectively. Subsequently, the medium was removed, and the cells were washed three times with PBS solution and fixed with 4% paraformaldehyde solution for 15 min. Then the cells were stained with DAPI (Invitrogen) diluted in PBS for 5 min at RT. Localization of DOX-loaded micelles and DOX in cells was visualized using the confocal microscope (Olympus Corporation).

The same procedure was carried out to show the lysosomes using LysoTracker Green DND-26 (Thermo Fisher Scientific). Cells were incubated for 2, 4, or 6 h at 37 °C. Hochest33342 (Thermo Fisher Scientific) was used to stain the nucleus for 30 min. Then the cells were washed with PBS and incubated with LysoTracker Probes for 1–5 minutes at 37 °C. All observations should pay attention to the protection of light.

Flow cytometry (ACEA BIO) was used to quantitatively detect the uptake of DOX-loaded micelles. 95D cells were seeded in 6-well plates at 3.0 × 10^5^ cells per well and incubated for 24 h. Subsequently, cells were incubated with DOX/DOX-loaded micelles for another 0.5 h, 2 h and 6 h, with a blank medium as the control group. After incubation, the cells were washed twice with cold PBS (pH 7.4) solution, and finally the cellular uptake efficiency of the DOX was quantified using flow cytometry.

### 2.9. *In vivo* antitumor efficacy

95D cells (7 × 10^6^ per mouse) in 100 μL were injected into the right flank of the mice. The tumor volume was measured daily. Mice were randomly divided into three groups when tumor volume reached about 100–150 mm^3^ (*n* = 5) to do the following experiment. Free DOX (5 mg kg^−1^), DOX-loaded micelles (DOX 5 mg kg^−1^) and saline were injected intravenously *via* the tail vein and repeated every 3 days. Tumor size was measured twice a week. The longest part of the tumor was the length and the narrowest part perpendicular to the length was the width. The body weight was measured to monitor the toxicity. The tumor volume was calculated by the following formula: tumor volume (mm^3^) = length × width^2^/2. At the end of the therapy, mice were anesthetized, tumors and major organs were excised for pathological examination.

### 2.10. Pharmacokinetics and biodistribution

Female BALB/c nude mice (6–8 weeks age) were randomly divided into two groups (*n* = 3): free DOX and DOX-loaded micelles at an identical dose of 10 mg kg^−1^ DOX. The mice were injected *via* the tail vein. At predetermined time points (5, 10, 30 min, and 1, 2, 4, 8, 12, 24 h), 0.5 mL of blood was collected into heparin-treated tubes from the retrobulbar vein and centrifuged at 6000 rpm for 5 min to obtain plasma samples. A linear standard curve of DOX was created and used for measuring the concentration of DOX in blood. Fluorescence of DOX was measured using a microplate reader (Biotek, USA) with excitation at 470 nm and emission at 590 nm. The major organs (heart, spleen, liver and kidney) were harvested at 24 h post injection. Then, the tissues were weighed and homogenized. The accumulation of DOX in the major organs and tumors were measured using the microplate reader.

DiD-loaded MPEG-PEI-PBLL were used to evaluate the *in vivo* distribution in tumor-bearing mice. The preparation of the micelles has been described above. Tumor-bearing mice were intravenously injected 100 μL DiD-loaded micelles *via* the tail vein and images of mice were acquired at 0 min, 20 min, 2 h, 6 h, 24 h, 48 h after administration. After 48 h, mice were anesthetized, tumors and major organs were excised and imaged using IVIS Spectrum (PerkinElmer). At trial conclusion, the mice were sacrificed and necropsied to harvest the main organs (heart, liver, spleen, lung, and kidney) and tumors for *ex vivo* fluorescence imaging.

## 3. Result and discussion

### 3.1. Synthesis of the copolymer

**Fig. 1:**
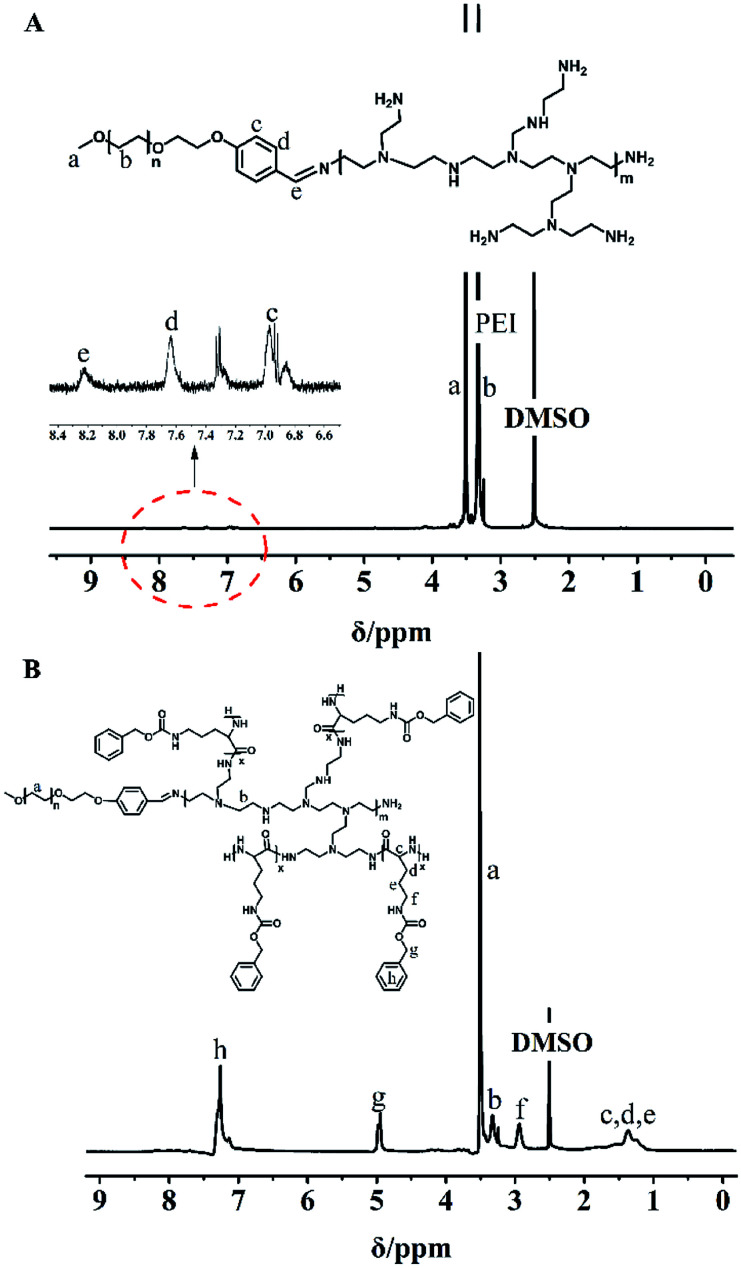
^1^H NMR spectra of (A) MPEG-PEI and (B) MPEG-PEI-PBLL in DMSO-*d*_6_.

MPEG-PEI-PBLL was synthesized through sequential ring opening polymerization initiated by MPEG-PEI. Fig. 1 showed ^1^H NMR of MPEG-PEI-PBLL in DMSO-*d*_6_ and each peak could find its corresponding structure, exhibited the polymer has been synthesized successfully. Using MPEG as reference, the ratio of the blocks (MPEG, PEI, BLL) was 1 : 1 : 11, which was approximately equal to the design value. In the FT-IR spectra of MPEG-PEI-PBLL (Fig. S1†), characteristic absorption peaks at 1690 (*ν*_C=O_) and 1415 (*ν*_C(O)–NH_) cm^−1^ were attributed to the amide bond on the copolymer backbone and the stretching of –C=N– bond was verified at 1520 cm^−1^, which further confirmed the molecular successful synthesis of MPEG-PEI-PBLL copolymers.

The weight of MPEG-PEI-PBLL was obtained by gel permeation chromatograph (GPC) (*M*_n_ = 5782, PDI = 1.56) (Fig. S2†).

### 3.2. Formation and pH-responsive ability of the micelles

**Fig. 2:**
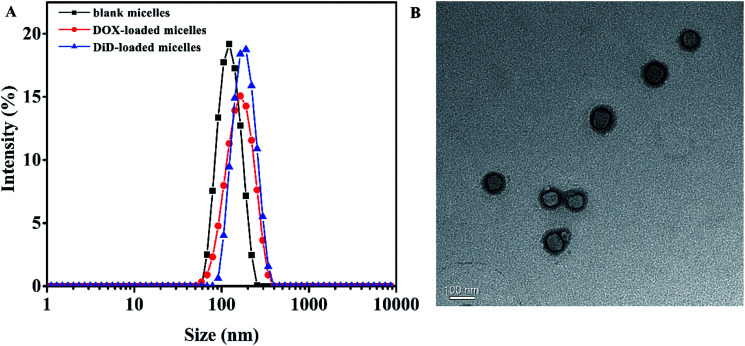
(A) Particle size of blank micelles, DOX-loaded micelles, and DiD-loaded micelles self-assembled by MPEG-PEI-PBLL; (B) TEM micrograph of blank MPEG-PEI-PBLL micelles.

In order to aggregate at tumor site, particle size of the micelles should meet the condition of Enhanced Permeability and Retention (EPR) effect,^53^ which was important for drug aggregating at the tumor site with no need of targeting modification. DLS measurements revealed that the blank micelles possess hydrodynamic diameters of approximately 117.6 nm with relatively narrow polydispersity index of 0.061 (Fig. 2A), and the particle size determined by TEM was around 88 nm (Fig. 2B), the difference might be attributed to the shrinkage of the micelles during the preparation of TEM sample, moreover obvious core–shell structure could be observed by TEM.

**Fig. 3:**
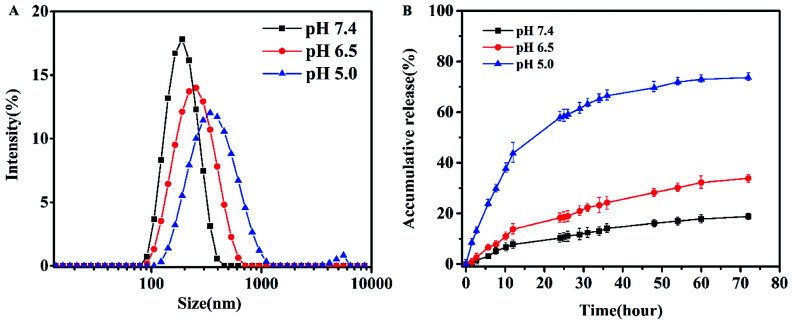
(A) Particle size of the micelles after dialysis in different pH buffer for 48 h at 37 °C. (B) *In vitro* DOX release in PBS for 72 h at 37 °C.

The CMC values of blank micelles of were measured to be 4 × 10^−3^ mg mL^−1^, the result was shown in Fig. S3,† implying the formation of MPEG-PEI-PBLL micelles in aqueous media at the concentration. It also indicated the micelles are stable in water and suitable to be drug carrier. As shown in Fig. S4B,† the blank micelles based on MPEG-PEI-PBLL broke up when the pH value reached 5.0, which was due to the cleavage of the Schiff base bond under acid condition. The stability of micelles in different pH was investigated (Fig. 3A), the results exhibited the particle size of micelles increased largely when the micelles were incubated at pH 5.0 for 48 h, indicated the micellar morphologies were influenced by variety of acidic environment. At pH 6.5, the variation of the particle size induced by change of pH was much smaller comparing with the change at pH 5.0. However, the particle size of micelles was changed slightly at pH 7.4 after 48 h incubation.

### 3.3. Encapsulation and release of DOX

DOX-loaded micelles were prepared by solvent exchange method. The particle size of DOX-loaded micelles increased a little (Fig. S4A†), but the polydispersity index was also close to 0.1, showing a unimodal distribution as measured by DLS at 25 °C. As shown in Table 1, DLC of the MPEG-PEI-PBLL micelles was measured to be 25.8% while the efficient was 87%. To our knowledge, most of the micelles that formed by linear polymer consist of PEG and polypeptide have the DLC lower than 15%. Therefore, the MPEG-PEI-PBLL micelles exhibited a great drug loading capacity due to the hyperbranched structure of the copolymer. DiD could also loaded into the micelles with high efficiency. Moreover, based on the cleavage of Schiff base linkages, the micelles could release drug at pH 5.0 with cumulative release about 78.4% in 72 h. Meanwhile, the micelles are stable at neutral environment, only 19.3% of DOX were released. It suggested the release behavior was greatly influenced by the acidic environment, these polypeptide micelles could respond to the change of pH values, which may be promising as nano drug carrier *in vivo*.

**Table 1:** Particle size and drug loading capacity of the micelles

Micelles	Size^a^	DLC^b^ (wt%)	DLE^b^ (%)	PDI^a^
Blank micelles	117.6	—	—	0.061
DOX-loaded micelles	159.3	25.8	87	0.134
DiD-loaded micelles	177.2	19.3	85	0.067

a Size and PDI were measured by DLS.

b Determined by UV absorption.

### 3.4. Cellular uptake behaviors of DOX-loaded micelles

**Fig. 4:**
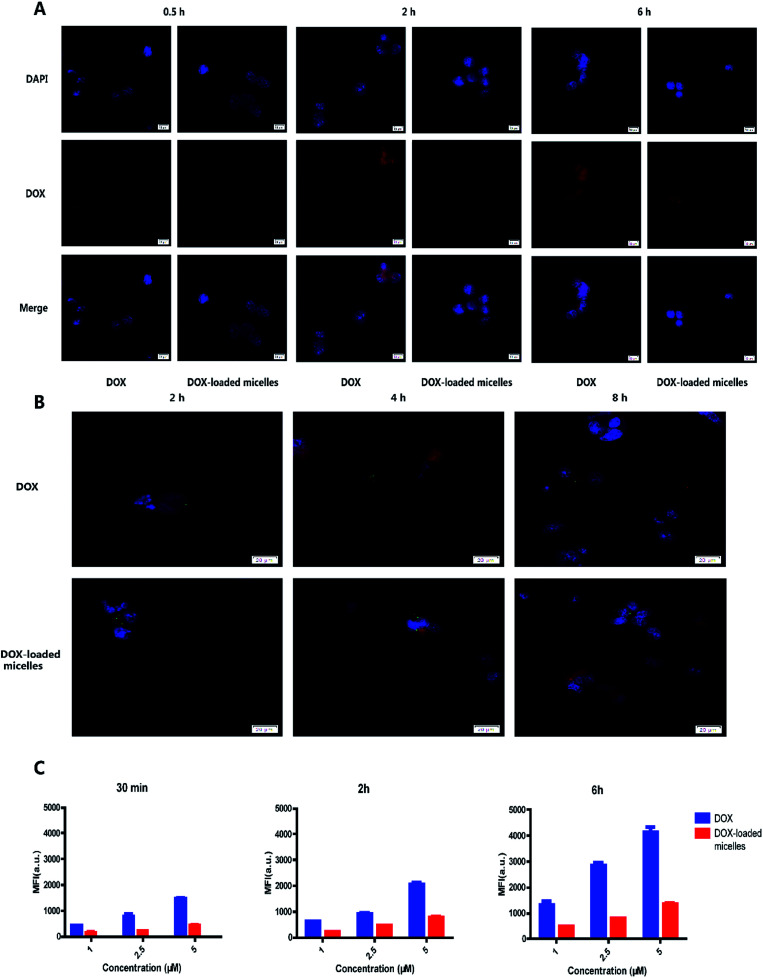
Cell uptake and intracellular distributions. (A) Confocal microscopy images of 95D cells after 0.5 h, 2 h and 6 h incubation with DOX or DOX-loaded micelles (red: DOX; blue: nuclei). (B) Confocal microscopy images of 95D cells incubated with DOX or DOX-loaded micelles for 2 h, 4 h and 8 h prior to imaging (green: lysosomes; red: DOX; blue: nuclei). The green fluorescence gradually became apparent over time and was more obvious in DOX-loaded micelles incubated cells. (C) Flow cytometry images of 96D cells incubated with different concentrations of DOX and DOX-loaded micelles.

The intracellular drug uptake was evaluated using confocal microscopy and flow cytometry. From the confocal microscopy images (Fig. 4A), 95D cells incubated with free DOX exhibited strong red fluorescence compared to the micelles group after 0.5 h incubation. After 2 h of incubation with DOX or DOX-loaded micelles, strong red fluorescence could be observed in nucleus and cytoplasm. Moreover, the intensity of green fluorescence from lysosomes was stronger in the DOX-loaded micelles group (Fig. 4B). It was known to us that rapid penetration of DOX into the cells would result in systemic toxicity *in vivo*. Moreover, the uptake profile in flow cytometry showed that more uptake profile at each time point (Fig. 4C), and the percentage of positive cells was shown in Fig. S5,† suggesting that DOX-loaded micelles were endocytosed slower than free DOX. These results demonstrated the micelles can avoid systemic toxicity effectively and reduce side effect.

### 3.5. Cell viability assays

**Fig. 5:**
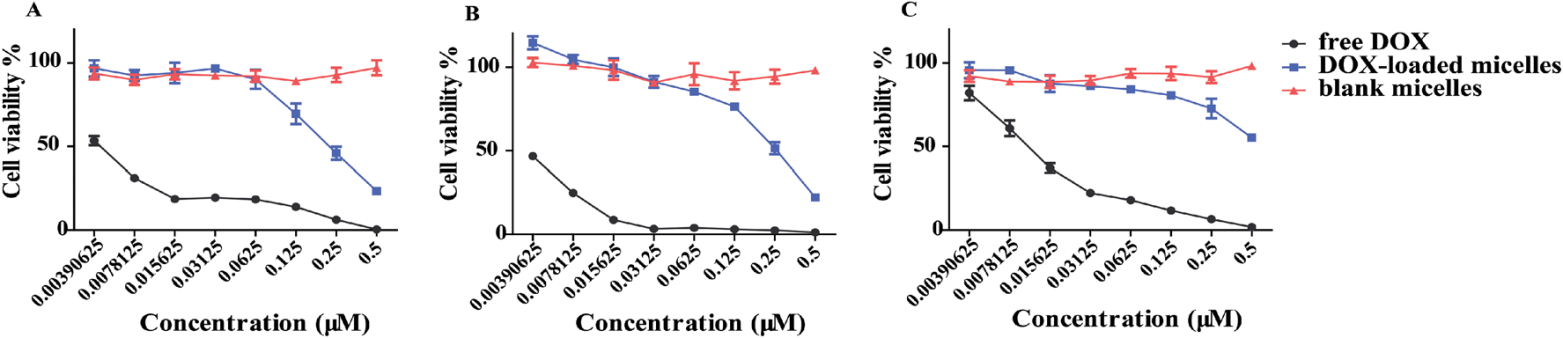
Cell viability of (A) 95D, (B) A549 and (C) H9C2 cells after 72 h incubation with DOX or DOX-loaded micelles determined by CCK-8 assay (n-3). IC_50_ values (μM) of DOX were calculated to be 0.003 for A549 and 95D, and 0.012 for H9C2. IC_50_ values (μM) of DOX-loaded micelles decreased to 0.229 for A549, 0.223 for 95D and 0.6 for H9C2, respectively. The concentration refers to the DOX concentration. Blank micelles concentration was calculated by the DLC value.

DOX-loaded micelles showed a lower influence in cell viability most likely due to lingeringly DOX uptake and release inside a cell. Free materials alone did not cause any cytotoxicity. An obviously decreased in cell viability was observed at lower concentrations of DOX-loaded micelles in 95D and A549 cells, except H9C2 cells (Fig. 5), which exhibited no significant difference. The results suggested the potential of DOX-loaded micelles could be used as an antitumor strategy. More importantly, cell viability assays revealed that the cytotoxicity of DOX reduced when the drug was encapsulated into the MPEG-PEI-PBLL micelles. The reason may be due to the difference in the uptake pathway. Free DOX could pass through the cell membrane easily with free diffusion. However, the DOX-loaded micelles can only be internalized by endocytosis, DOX was released from the carriers, and then entered the nucleus to exert their activity.

### 3.6. *In vivo* biodistribution

**Fig. 6:**
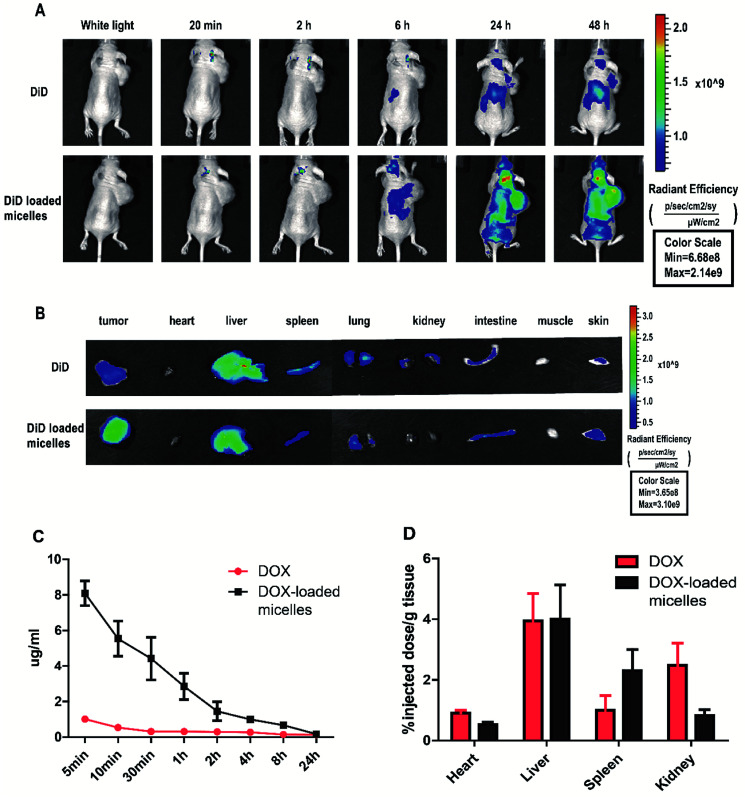
The biodistribution and tumor targeting of DiD-loaded micelles in 95D tumor-bearing mice *via* the near infrared optical imaging (NIRF) approach. (A) After intravenously injection of free DiD or DiD-loaded micelles, images of 95D tumor-bearing mouse at different time points and (B) *ex vivo* images of dissected tumor and major organs obtained at 48 h after injection. Pharmacokinetics and biodistribution of DOX and DOX-loaded micelles *in vivo*. (C) DOX concentration in the serum (*n* = 3) over 24 hours (D) DOX accumulation in organs (*n* = 3) after 24 h.

NIRF dye DiD was used to exhibit the distribution of drug delivery system *in vivo*. Free DiD and DiD-loaded micelles (with an equal amount of DiD) were injected intravenously into mice and pictures were taken at predetermined time interval. It was found the fluorescence intensity of DiD in tumors increased gradually and reached maximum at 48 h after treatment with DiD-loaded micelles (Fig. 6A). On the contrary, little amount of tumor fluorescence could be detected in mice treated with DiD. After the drug injected 48 hours, tumors and major organs were excised for *ex vivo* imaging. As shown in Fig. 6B, the tumor in DiD-loaded micelles group exhibited significantly strong fluorescence intensity compared to free DiD. In addition, the liver showed relatively stronger fluorescence intensity than the rest normal organism in both groups, which was due to the elimination of macromolecules *via* the reticuloendothelial system (RES). Pharmacokinetic parameters were showed in Table S1.† The *ex vivo* images further confirmed DiD-loaded micelles could preferentially accumulate in tumor compared to mice treated with free DiD. The results implied that the micelles could protect the agents from being eliminated through prolonging its blood circulation time, and the micelles could accumulate at tumor site passively *via* EPR effect.

To further study the drug aggregation in the tissue *in vivo*, blood samples were collected from nude mice treated with DOX or DOX-loaded micelles at predetermined post-injection time points and DOX concentration. DOX-loaded micelles have a significantly prolonged circulation in blood as shown in Fig. 6C, it could be attributed to the stability of the micelles at pH 7.4, the pH of bloodstream. The cardiotoxicity of DOX was well-known, however, DOX-loaded micelles demonstrated lower cytotoxicity than free DOX, suggesting that DOX-loaded micelles may reduce cardiac toxicity *in vivo*. The major organs of the mice were excised in the end, drug accumulation was studied. Fluorescence signals of DOX-loaded micelles in kidney and heart were lower compared to free DOX (Fig. 6D), showing the side effects of drug loading micelles to other organs were much more slight, comparing to the free DOX.

### 3.7. Therapeutic study *in vivo*

**Fig. 7:**
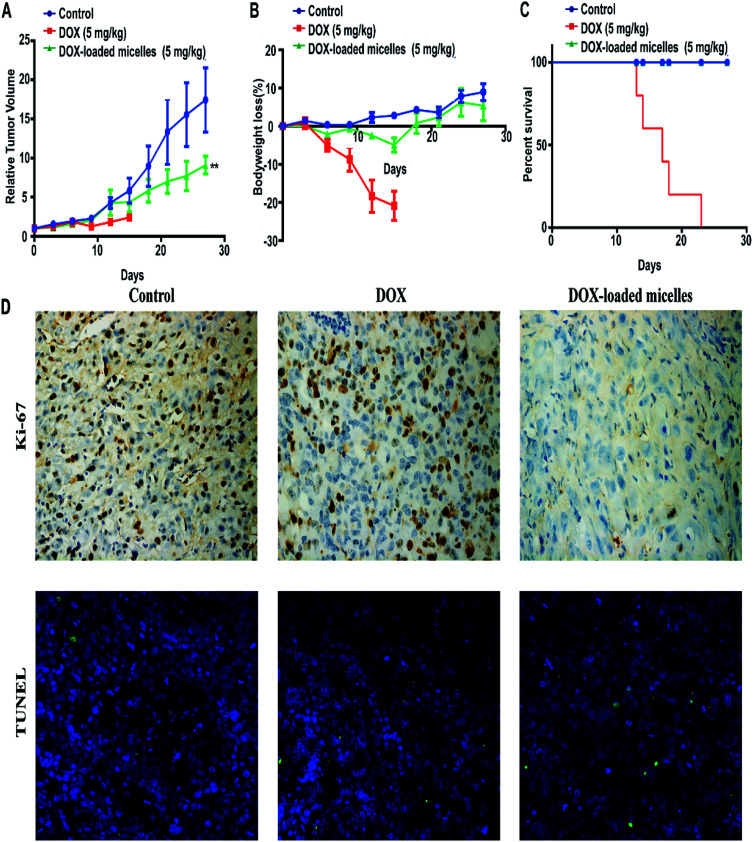
95D tumor-bearing mice were divided into three groups (*n* = 5) and intravenously injected with saline, DOX (5 mg kg^−1^) and DOX-loaded micelles (5 mg kg^−1^) every 3 days. (A) Relative tumor volume growth. (B) Bodyweight loss. (C) Survival rate of mice. Data expression were presented as mean ± SD. **P < 0.01. (D) TUNEL and Ki-67 staining of tumor tissues (×100) treated with DOX-loaded micelles, DOX, and saline. The green fluorescence in the images indicate apoptosis cells. The brown colors in the images indicate Ki-67 positive.

**Fig. 8:**
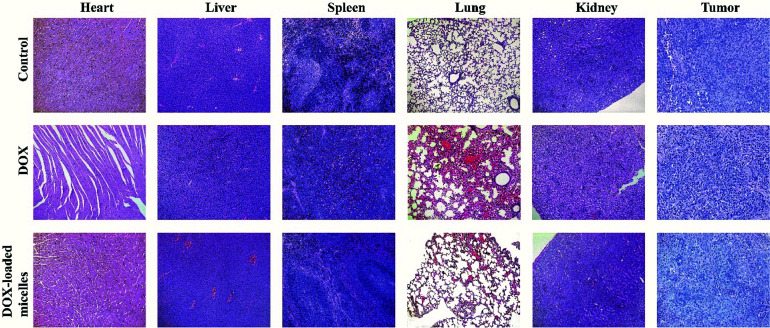
Histological analysis for major organs (×100) of mice administrated with saline, DOX, and DOX-loaded micelles.

Subcutaneous lung cancer xenograft model was introduced to investigate the therapeutic efficiency and toxicity of DOX-loaded micelles. As shown in Fig. 7A, tumor growth was inhibited after being treated with DOX-loaded micelles comparing to the control group, and no significant change in bodyweight loss and survival rate were observed in both groups. However, mice treated with free DOX demonstrated a dramatic decline in bodyweight loss and died within 23 days (Fig. 7B). To further investigate the *in vivo* toxicity, hematoxylin and eosin (H&E) staining was conducted (Fig. 8). After DOX-loaded micelles treatment, a large area of cancer tissue necrosis could be observed obviously, implying the apoptosis induced by DOX-loaded micelles led to the distinct inhibition of tumor growth. The major organs were harvested from the mice after treated with drug loading micelles. The H&E staining of these organs confirmed there were no apparent histopathological abnormalities, degenerations, or lesions compared to those treated with saline. As shown in Fig. 7D, TUNEL results showed that much more apoptosis occurred in DOX-loaded micelles group compared to the other two groups, it was consistent with H&E results. The Ki-67 immunohistochemical staining could reflect the degree of proliferation and malignancy of cells. As shown in Fig. 7D, comparing to other two groups, much fewer Ki-67 positive cells could be observed in the drug loading group. DOX-loaded micelles exhibited great tumor growth inhibition and improved livability of mice because the micelles had longer blood circulation time, slower drug release rate and good targeting ability to tumors. Meanwhile, DOX-loaded micelles reduced cardiotoxicity significantly compared to free DOX owing to more efficient delivery to the tumor site. Compared with the control group, the bodyweight loss and pathological examinations of other normal organs showed no differences after treatment with DOX-loaded micelles, which further confirmed its better biosafety. Moreover, H&E stained sections showed pyknosis of nuclei and condensation of the cytoplasm in tumor after administration of the drug loading micelles and more necrotic cardiocyte was observed in DOX loading group. These results confirmed the micelles possess the potential as safe and effective drug loading vehicles for tumor therapy applications.

## 4. Conclusions

In summary, pH-responsive polymer micelles were successfully prepared based on triblock copolymer MPEG-PEI-PBLL. The pH-triggered cleave of Schiff base linkages in the polymer endow the micelles with the ability to respond to the change of environmental acidic change. Meanwhile, the drug loading capacity is enhanced due to the hyperbranched structure of polymer. The cellular uptake studies showed that the DOX encapsulated in micelles have a much slower uptake rate compared to free DOX, which can decrease systemic toxicity *in vivo*. *In vivo* biodistribution demonstrates the micelles can effectively reduce excessive deleterious effects on the healthy cell and tissues. Moreover, *in vivo* therapeutic and pharmacokinetics studies suggest the micelles can accumulate at the tumor site and inhibit the tumor growth while improve the livability of mice. These results demonstrate that this hyperbranched pH-responsive copolymer is a promising material for drug-loaded carriers.

## Conflicts of interest

There are no conflicts to declare.

## Supplementary Material

RA-010-D0RA01241F-s001
