# Supplementary material for: pH-Responsive hyperbranched polypeptides based on Schiff bases as drug carriers for reducing toxicity of chemotherapy
Source: RSC Adv. 2020 Apr 6;10(23):13889–99. doi: 10.1039/d0ra01241f (PMC9051653; doi:10.1039/d0ra01241f)
Supplement: RA-010-D0RA01241F-s001 [file RA-010-D0RA01241F-s001.pdf]

Electronic Supplementary Material (ESI) for RSC Advances.
This journal is © The Royal Society of Chemistry 2020

Supplementary Information

pH-Responsive Polypeptides nanoparticles based on Schiff base for efficient drug encapsulation and cancer therapy

Rui Yan1, Xinyi Liu1, Junjie Xiong, Qiyi Feng, Junhuai Xu, Haibo Wang*, Kai Xiao*

West China Hospital; College of Biomass Science and Engineering, Sichuan University, Chengdu, 610065, China.

Correspondence E-mail whb6985@scu.edu.cn; xiaokaikaixiao@scu.edu.cn

[1]R. Yan and X. Liu contributed equally to this work.

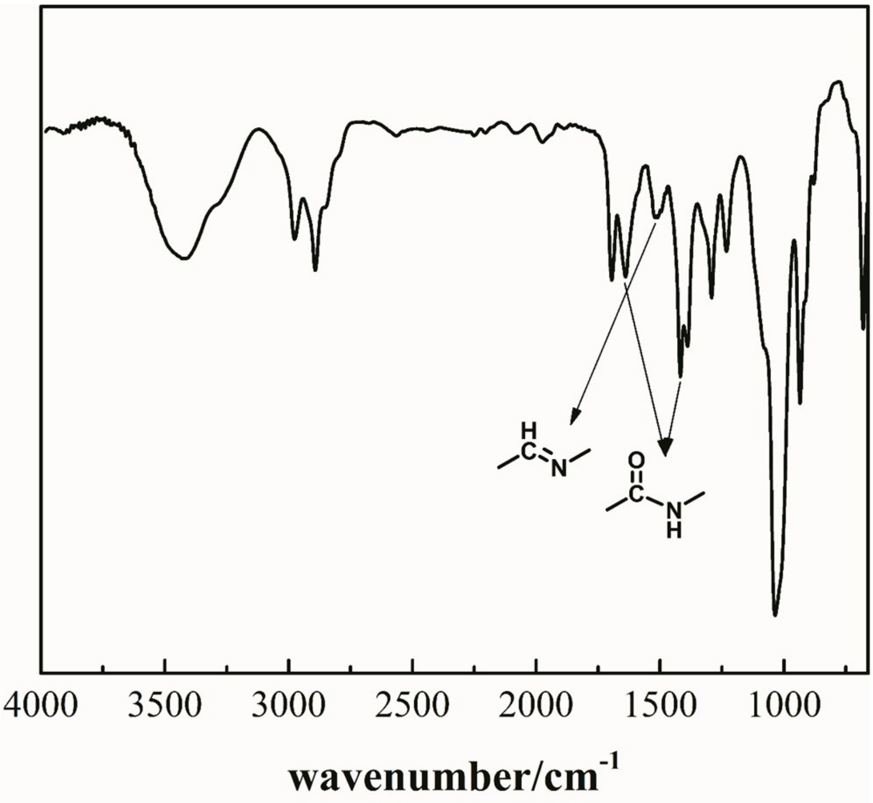

Figure S1. FT-IR spectra of MPEG-PEI-PBLL

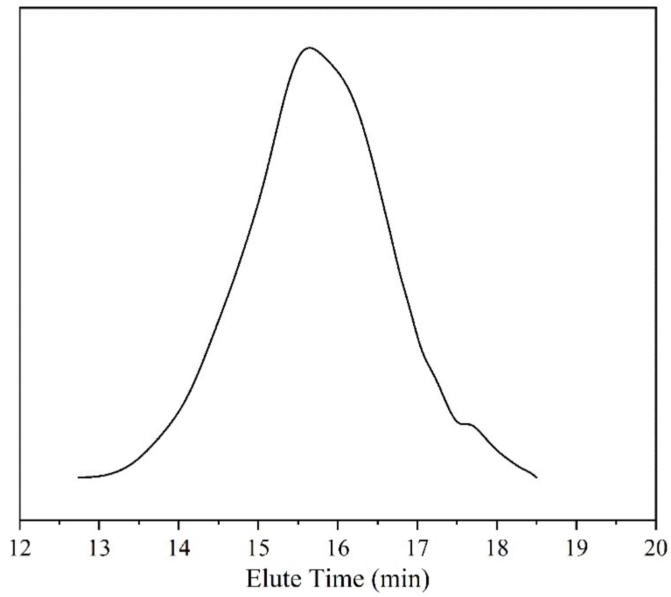

Figure S2. GPC profiles of MPEG-PEI-PBLL (Elution: DMF, PS standards).

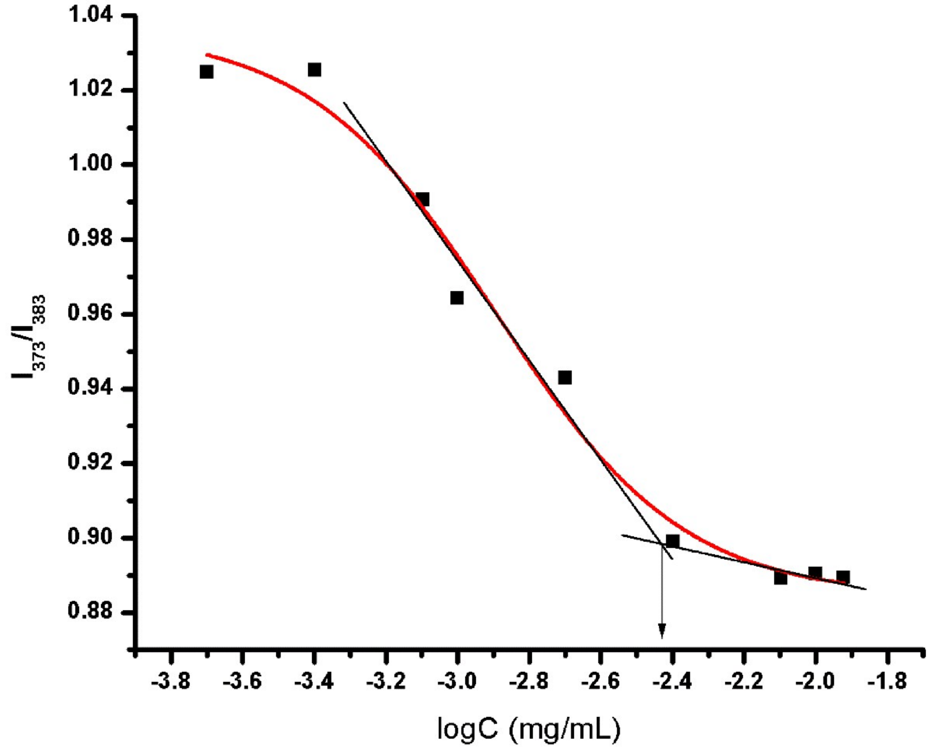

Figure S3. Critical micelle concentration (CMC) of the MPEG-PEI-PBLL micelles

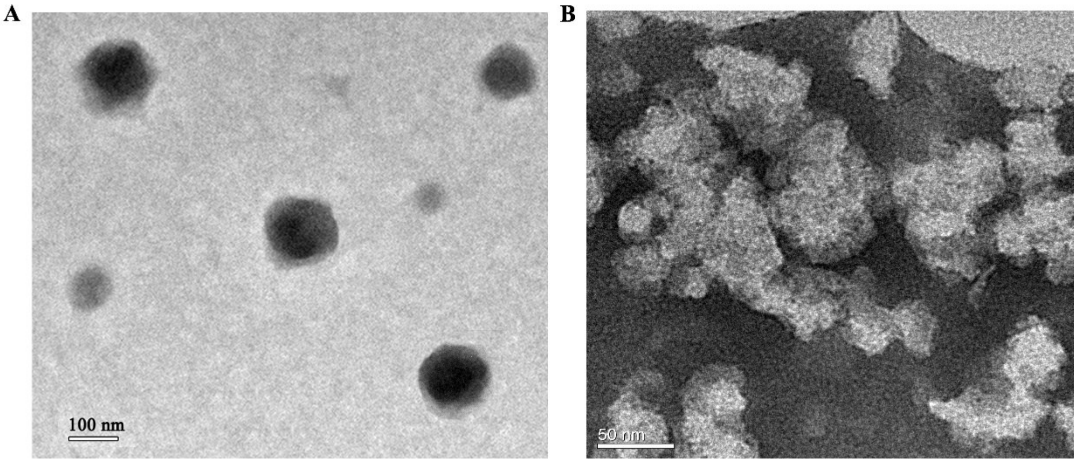

Figure S4. TEM images of (A) DOX-loaded micelles and (B) blank micelles incubated in PBS at pH 5.0 after 48 h.

Table S1 Pharmacokinetic parameters of BALB/c female mice after intravenous administration of DOX and the DOX-loaded micelles at doses of 10 mg DOX/kg (n = 3).

Parameters	Free DOX	DOX-loaded micelles
$AUC_{0\text{-}24}$ (μg/mL*h)	4.51±0.67	19.53±1.72
CL (mL/h/kg)	255.96±70.51	1212.33±468.00
Cmax (mg/L)	2.08±0.91	12.12±3.02

AUC: area under the time concentration curve; CL: clearance; C_{max}: peak concentration

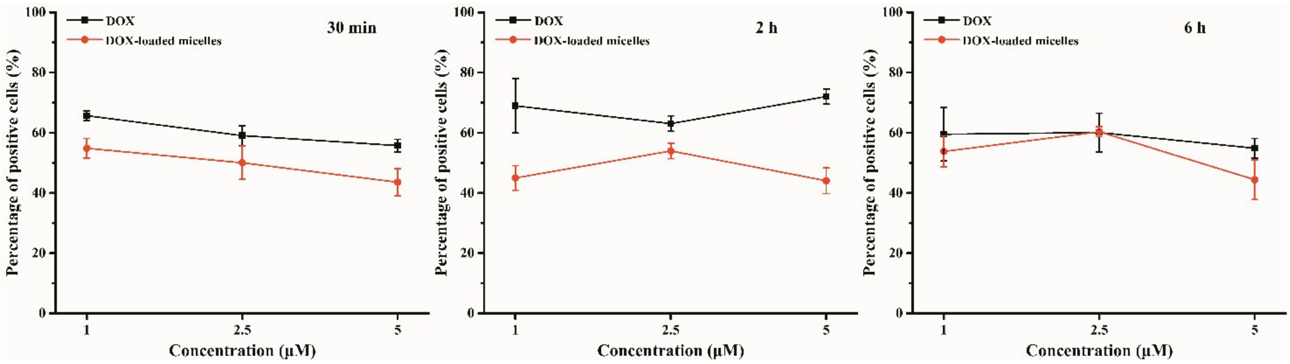

Figure S5 the percentage of positive cells after incubated with DOX and DOX-loaded micelles in different concentration and time
